# Drug, Opioid-Involved, and Heroin-Involved Overdose Deaths Among American Indians and Alaska Natives — Washington, 1999–2015

**DOI:** 10.15585/mmwr.mm6750a2

**Published:** 2018-12-21

**Authors:** Sujata Joshi, Thomas Weiser, Victoria Warren-Mears

**Affiliations:** ^1^Northwest Portland Area Indian Health Board, Northwest Tribal Epidemiology Center, Portland, Oregon; ^2^Portland Area Office, Indian Health Service, Portland, Oregon.

## Abstract

The opioid epidemic has resulted in a threefold increase in drug overdose deaths in the United States during 1999−2015 (*1*). Whereas American Indians/Alaska Natives (AI/AN) have experienced larger increases in drug overdose mortality than have other racial/ethnic groups in the United States (*2*), little is known about the regional impact of opioids in tribal and urban AI/AN communities. To address this data gap, death records from the Washington State Center for Health Statistics, corrected for misclassification of AI/AN race, were examined to identify trends and disparities in drug, opioid-involved, and heroin-involved overdose mortality rates for AI/AN and non-Hispanic whites (whites) in Washington. Although AI/AN and whites had similar overdose mortality rates during 1999–2001, subsequent overdose rates among AI/AN increased at a faster rate than did those among whites. During 2013–2015, mortality rates among AI/AN were 2.7 and 4.1 times higher than rates among whites for total drug and opioid-involved overdoses and heroin-involved overdoses, respectively. Washington death certificates that were not corrected for misclassification of AI/AN race underestimated drug overdose mortality rates among AI/AN by approximately 40%. National statistics on the opioid epidemic, which report that overdose mortality rates are significantly higher among whites than among AI/AN, are not reflective of regional prevalences, disparities, and trends. Comprehensive efforts to address the opioid epidemic in AI/AN communities rely on strong partnerships between tribal governments and local, state, and federal entities. Additional measures are needed for community-based surveillance, treatment, and prevention to effectively respond to the epidemic across diverse tribal and urban AI/AN communities.

Washington drug overdose deaths were identified using death certificate statistical files for 1999–2015 from the Washington State Center for Health Statistics. Death certificates were corrected for misclassification of AI/AN race by conducting probabilistic record linkages between Washington death certificates and the Northwest Tribal Registry (a database of personal identifiers for AI/AN patients seen in IHS, tribal, and urban Indian health clinics in Idaho, Oregon, and Washington) (*3*). Washington death certificates were matched to the Northwest Tribal Registry using social security number, date of birth, name (last, first, and middle), and sex. Two staff members conducted clerical review of all potential matched pairs to identify true matches. AI/AN decedents included those with any mention of American Indian or Alaska Native background (regardless of Hispanic ethnicity) in the multiple race fields on the death certificate and those who matched with the Northwest Tribal Registry database but had no indication of AI/AN background on the death certificate (i.e., misclassified AI/AN records). AI/AN were compared with the majority white population to identify relative disparities in Washington. Uncorrected national and state-level estimates for 2013–2015 were obtained from the CDC WONDER Online Database for comparison.*

For both corrected and uncorrected data, total drug overdose deaths were identified as deaths with one of the following *International Classification of Disease, Tenth Revision* (ICD-10) codes for drug poisoning in the underlying cause of death field on the death record: X40–X44 (accidental poisoning by and exposure to drugs), X60–X64 (intentional self-poisoning by and exposure to drugs), X85 (assault by drugs), or Y10–Y14 (poisoning by and exposure to drugs, undetermined intent). Opioid-involved overdose deaths include the subset of drug overdose deaths with at least one of the following ICD-10 codes in the multiple cause of death fields: T40.0 (opium), T40.1 (heroin), T40.2 (other natural or semisynthetic opioids), T40.3 (methadone), T40.4 (other synthetic opioids), or T40.6 (other and unspecified narcotics). Heroin-involved overdose deaths include the subset of drug overdose deaths with heroin (ICD-10 code T40.1) listed in any multiple cause of death field. Trends were calculated as 3-year rolling averages of age-adjusted mortality rates during the period 1999–2015. Rates were age-adjusted to the U.S. 2000 standard population using National Center for Health Statistics (NCHS) vintage 2015 bridged race estimates as population denominators. For rates among AI/AN, 95% confidence intervals (CIs) were based on the gamma distribution to account for small cell sizes (*4*), and CIs for rates among whites were calculated using the normal approximation method. Metropolitan and nonmetropolitan counties were designated using the NCHS 2013 Urban-Rural Classification Scheme for Counties (*5*).^†^ Link Plus v.2.0 was used to conduct the probabilistic record linkages, and statistical software was used to analyze the corrected Washington death certificates. Uncorrected drug and opioid-involved overdose counts, rates, and CIs for the United States and Washington were obtained using Multiple Cause of Death Data from the CDC WONDER online database (https://wonder.cdc.gov/mcd.html).

During 1999–2001, based on death certificates corrected for AI/AN misclassification, AI/AN and whites in Washington had similar age-adjusted total drug, opioid-involved, and heroin-involved overdose mortality rates (Figure). Overdose death rates increased significantly for both groups in subsequent years; however, the increase was much sharper among AI/AN than among whites. During 2013–2015, 184 drug overdose deaths occurred among AI/AN in Washington, including 126 (68.5%) that involved opioids. The rates were higher for total drug (2.7 times), opioid-involved (2.7), and heroin-involved overdose mortality (4.1) among AI/AN than among whites (Table 1). Among AI/AN in Washington, the total drug overdose rate among males was 1.7 times that among females (Table 2). AI/AN aged 25–54 years had higher rates of drug overdose mortality than did those in younger and older age groups. Age-specific drug overdose mortality rates among AI/AN were almost twice those among whites. The majority of drug overdose deaths among AI/AN and whites occurred among Washington residents living in metropolitan (urban) counties. Among whites, similar rates of drug overdose deaths occurred among urban and rural residents; the overdose death rate among urban-dwelling AI/AN was 1.4 times that of AI/AN living in rural areas, although this difference was not statistically significant. The demographic distributions for opioid-involved and heroin-involved overdose deaths were similar to those observed for total drug overdose deaths.

**FIGURE:**
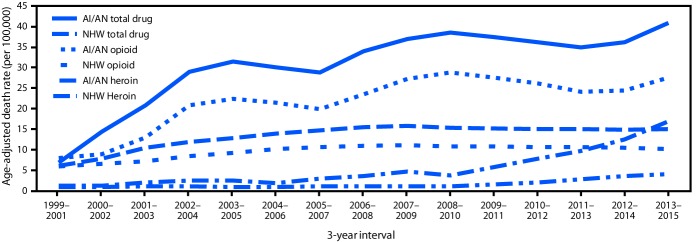
Age-adjusted death rates*^,†^ for total drug,**^§^** opioid-involved, and heroin-involved overdose deaths among American Indians/Alaska Natives and non-Hispanic whites — Washington, 1999–2015 **Source:** Washington Center for Health Statistics Death Files 1999–2015, corrected for AI/AN misclassification through linkage with the Northwest Tribal Registry. **Abbreviations:** AI/AN = American Indian/Alaska Native; NHW = non-Hispanic white. * Per 100,000 persons. ^†^ Three-year rolling averages. ^§^ Total drug overdose deaths include opioid-involved and nonopioid-involved deaths; opioid-involved deaths include heroin-involved deaths.

**TABLE 1:** Corrected* and uncorrected age-adjusted total drug,^†^ opioid-involved, and heroin-involved overdose mortality rates (per 100,000 population) and rate ratios for American Indians/Alaska Natives and non-Hispanic whites — Washington and United States, 2013–2015

Race	Population	Type of drug overdose rate (95% CI)		
		Total drug^†^	Opioid-involved	Heroin-involved
**American Indian/Alaska Native**	WA (corrected)	40.9 (35.1–48.0)	27.5 (22.8–33.5)	16.7 (13.1–21.6)
	WA (uncorrected)	28.7 (23.7–33.7)	19.6 (15.7–24.2)	11.9 (8.9–15.5)
	US (uncorrected)	13.2 (12.5–13.8)	7.6 (7.1–8.0)	2.4 (2.1–2.6)
**White, non-Hispanic**	WA (corrected)	15.1 (14.5–15.7)	10.2 (9.7–10.7)	4.1 (3.7–4.4)
	WA (uncorrected)	15.7 (15.0–16.3)	10.6 (10.1–11.2)	4.3 (4.0–4.6)
	US (uncorrected)	19.2 (19.1–19.3)	12.1 (12.0–12.2)	4.4 (4.4–4.5)
**AI/AN:NHW rate ratios**				
WA AI/AN:NHW (corrected)	—	2.7 (2.3–3.1)	2.7 (2.3–3.2)	4.1 (3.2–5.2)
WA AI/AN:NHW (uncorrected)	—	1.8 (1.3–2.6)	1.8 (1.5–2.3)	2.8 (2.1–3.6)
U.S. AI/AN:NHW (uncorrected)	—	0.69 (0.65–0.72)	0.63 (0.59–0.67)	0.55 (0.49–0.61)
WA AI/AN (corrected:uncorrected)	—	1.4 (1.0–2.1)	1.4 (1.1–1.8)	1.4 (1.0–2.0)

**Sources:** Washington Center for Health Statistics Death Files 2013–2015 linked with the Northwest Tribal Registry (corrected data); CDC WONDER online database, Multiple Cause of Death data 2013–2015 (uncorrected data).

**Abbreviations:** AI/AN = American Indian/Alaska Native; CI = confidence interval; NHW = non-Hispanic white; WA = Washington.

* Data are corrected for misclassification of AI/AN race through probabilistic record linkage with the Northwest Tribal Registry.

^†^ Total drug overdose deaths include opioid-involved and nonopioid-involved deaths; opioid-involved deaths include heroin-involved deaths.

**TABLE 2:** Number and age-adjusted rates (per 100,000 population) of total drug overdose deaths for American Indians/Alaska Natives and non-Hispanic whites, by sex, age, and rural/urban residence — Washington, 2013–2015

Characteristic	American Indian/Alaska Native			Non-Hispanic white		
	No.	Rate (95% CI)	Rate ratio (95% CI)	No.	Rate (95% CI)	Rate ratio (95% CI)
Sex						
Male	116	51.8 (42.7–64.7)	1.7 (1.3–2.3)	1,422	17.6 (16.6–18.5)	1.4 (1.3–1.5)
Female	68	30.1 (23.3–39.2)	Referent	1,040	12.5 (11.7–13.4)	Referent
**Age group (yrs)**						
<25	18	8.4 (5.0–13.2)	Referent	157	3.7 (3.1–4.3)	Referent
25–39	59	57.0 (43.4–73.5)	6.8 (4.0–11.5)	628	20.8 (19.2–22.5)	5.6 (4.8–6.8)
40–54	76	89.7 (70.7–112.3)	10.7 (6.4–17.9)	974	30.8 (28.9–32.8)	8.3 (7.1–10.0)
≥55	31	39.4 (26.8–55.9)	4.7 (2.6–8.4)	703	14.4 (13.4–15.5)	3.9 (3.3–4.7)
**County type of residence**						
Metropolitan (urban)	160	43.3 (36.7–51.5)	1.4 (0.9–2.2)	2,195	15.9 (14.0–17.8)	1.1 (0.9–1.2)
Nonmetropolitan (rural)	24	30.5 (19.3–48.1)	Referent	267	15.0 (14.3–15.7)	Referent

**Source:** Washington Center for Health Statistics Death Files 2013–2015, corrected for AI/AN misclassification through linkage with the Northwest Tribal Registry.

**Abbreviation**: CI = confidence interval.

During 2013–2015, based on CDC WONDER data uncorrected for AI/AN misclassification, in the United States, AI/AN had lower total drug, opioid-involved, and heroin-involved overdose mortality rates than those among whites (Table 1). Even before correction for AI/AN misclassification, AI/AN in Washington had higher drug, opioid-involved, and heroin-involved overdose mortality rates than did whites in Washington and AI/AN in the United States. Compared with Washington death certificates corrected for AI/AN misclassification, CDC WONDER data underestimated overdose mortality counts and rates among AI/AN in Washington by approximately 40% (Table 1).

## Discussion

Since 1999, the rate of increase in drug, opioid-involved, and heroin-involved overdose deaths among AI/AN in Washington has outpaced that among whites. In recent years, AI/AN in Washington experienced total drug and opioid-involved overdose mortality rates that were 2.7 times higher than those of whites in the state. The prevalence and disparity experienced among AI/AN in Washington differ from overdose mortality patterns observed at the national level, which indicate that U.S. whites experience significantly higher mortality rates from drug, opioid-involved, and heroin-involved overdoses than do U.S. AI/AN (Table 1).

AI/AN communities experience high rates of physical, emotional, and historical trauma and significant socioeconomic disparities, which might contribute to higher rates of drug use in these communities (*5*). AI/AN also face barriers to receiving quality medical and behavioral health care, resulting in part from longstanding underfunding of the Indian Health Service (IHS), tribal, and urban Indian clinics, as well as stigma associated with accessing behavioral health care in some communities (*6*). The differences in corrected and uncorrected rate estimates demonstrate the importance of accurately recording race on death certificates. Without the probabilistic linkage correction, uncorrected Washington death certificates underestimated overdose mortality rates among AI/AN by 40%. Misclassification of AI/AN in public health data can obscure the prevalence of disease and result in suppression of health statistics because of small numbers, which could affect the ability of state and federal programs to direct resources needed for a robust public health response to this epidemic.

The findings in this report are subject to at least six limitations. First, not all AI/AN in Washington seek care at IHS, tribal, or urban Indian health facilities, and thus, they would not have been included in the linkage. The Northwest Tribal Registry is known to underrepresent persons living in urban areas (*7*). Therefore, the actual number of drug overdose deaths and corresponding mortality rates among AI/AN might be higher than those reported in this analysis. Second, human error and bias might have been introduced during the probabilistic linkage process, particularly during clerical review of matched record pairs. Although double clerical review was employed as a strategy to decrease the introduction of bias, the possibility remains that human error could have resulted in the underascertainment or overascertainment of misclassified AI/AN records. Third, the NCHS bridged race estimates used as population denominators are known to inflate the Hispanic AI/AN population in the United States and therefore, result in the underestimation of mortality rates among AI/AN that include Hispanic AI/AN (*8*). Fourth, the circumstances under which toxicologic testing for drugs occurs and the testing methods themselves have changed over time (*1*), and these changes might account for some of the observed increases in drug and opioid-involved overdose deaths. Fifth, some heroin-involved deaths might have been misreported as morphine-involved deaths because of the similarity in metabolism of these two substances (*1*). Finally, this analysis of linkage-corrected death certificates was restricted to one state, which limits the generalizability of findings to AI/AN in other states.

Efforts that address the opioid epidemic are underway in tribal and urban AI/AN communities throughout the United States and rely on strong partnerships between tribal governments, regional Indian health boards, IHS and other federal agencies, tribal epidemiology centers, and local and state governments. IHS is addressing the epidemic in clinical settings through new prescribing policies, education for providers, and increased access to medication-assisted treatment and naloxone for first responders, in partnership with the Bureau of Indian Affairs (*9*). Additional efforts are needed for community-based surveillance, treatment, and prevention that address the variability in substance use disorder risk factors and outcomes across tribal and urban AI/AN communities. Programs that incorporate evidence-based strategies while addressing the diverse cultures, resources, and priorities of AI/AN communities might prove most effective in addressing current and future drug epidemics (*5*).

**Summary**

**What is already known about this topic?**

Nationally, American Indians and Alaska Natives (AI/AN) have experienced the largest increases in drug and opioid-involved overdose mortality rates compared with other racial/ethnic groups. Misclassification of AI/AN race is known to underestimate AI/AN mortality rates.

**What is added by this report?**

During 2013–2015, total drug and opioid-involved overdose mortality rates for AI/AN were 2.7 times higher than those of whites in Washington. Misclassification of AI/AN race in death certificates underestimated Washington AI/AN overdose mortality by approximately 40%.

**What are the implications for public health practice?**

Probabilistic linkages to correct misclassified race can improve accuracy of data on drug overdose mortality for AI/AN in Washington, which is important for state and federal resource allocation and program direction. Additional efforts are needed for community-based substance-use disorder surveillance, treatment, and prevention in AI/AN communities.

## Footnotes

* Data are from NCHS Multiple Cause of Death Files, 1999–2015, as compiled from data provided by the 57 vital statistics jurisdictions through the Vital Statistics Cooperative Program. https://wonder.cdc.gov/ucd-icd10.html.

† https://www.cdc.gov/nchs/data/series/sr_02/sr02_166.pdf.
